# Clinical efficacy of budesonide combined with acetylcysteine in the treatment of mycoplasma pneumonia infection

**DOI:** 10.1002/iid3.1068

**Published:** 2023-11-22

**Authors:** Jing Chen, Ying Zhu, Chunfeng Zheng, Wei Zhao, Qi Liu

**Affiliations:** ^1^ Department of Pediatrics The Second Affiliated Hospital of Qiqihar Medical University Qiqihar Heilongjiang China; ^2^ The Research Institute of Medicine and Pharmacy Qiqihar Medical University Qiqihar Academy of Medical Sciences Qiqihar Heilongjiang China

**Keywords:** budesonide/N‐acetylcysteine combination, inflammatory cytokines, mycoplasma pneumoniae pneumonia, therapy efficacy

## Abstract

**Objective:**

Mycoplasma pneumoniae pneumonia (MPP) is a common respiratory tract infectious disease in children. The study aimed to elucidate the therapeutic efficacy of aerosolized budesonide and N‐acetylcysteine combination therapy for MP infection in children.

**Methods:**

One hundred and twenty children with MP infection were included and divided into the control group (received aerosol inhalation of budesonide) and the experimental group (aerosolized budesonide and N‐acetylcysteine). After treatment, the disappearance time of clinical symptoms and efficacy were contrasted between the two groups.

**Results:**

With the passage of treatment time, the children's cough score of the two groups were gradually reduced. The children in the experimental group got well from the cough faster than the control group, and the difference reached a significant level on the 5th and 7th days. The time required for fever, rale, and cough to disappear in the experimental group was shorter than those in the control group. As the treatment progressed, a gradual decrease in serum interleukin‐6, tumor necrosis factor‐α, and C‐reactive protein values was detected in both groups, and the decrease was more significant in the experimental group. The total effective rate of the experimental group was 98.33%, which surpassed the control group (93.33%).

**Conclusion:**

Budesonide and N‐acetylcysteine combination therapy in the treatment of MP infection in children has a significant effect, and can quickly relieve the clinical symptoms of children with good safety. It is worthy of widespread clinical use.

## Graphical abstract

Budesonide and N‐acetylcysteine combination therapy in the treatment of mycoplasma pneumoniae infection in children has a significant effect, and can quickly relieve the clinical symptoms of children with good safety. It is worthy of widespread clinical use.

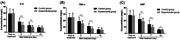

## 1 INTRODUCTION

Mycoplasma pneumoniae pneumonia (MPP) is a common respiratory tract infectious disease in children.^1^ Mycoplasma pneumoniae (MP) infection is the main cause of MPP in children.^2^ It is mainly transmitted by droplets with a high incidence in autumn and winter, and has certain latent mycoplasma pneumonia in children.^3^ Mycoplasma pneumoniae belongs to a pathogenic microorganism, it can attach to respiratory epithelial cells and damage respiratory mucosa to a certain extent.^4^, ^5^ In addition, infection with mycoplasma pneumoniae can trigger an immune response, promote the excessive release of inflammatory cytokines, and then induce an inflammatory response in the respiratory tract.^6^ The inflammatory response can also stimulate cough receptors, causing chronic cough, which will have adverse effects on children's learning and life.

Clinically, glucocorticoids are commonly applied to the treatment of MPP.^7^ Budesonide is a common medicine in the treatment of respiratory diseases.^8^ It is a glucocorticoid with a high local anti‐inflammatory effect and can strengthen the endothelium smooth muscle cell stability.^9^ At the same time, it can suppress inflammatory immune response and reduce antibody synthesis.^10^ Budesonide has a wide range of actions and few side effects, and the aerosol inhalation method enhances its comfort and safety.^11^ It can reclaim the imbalance of cytokine balance and ameliorate lung function.^12^ Recently, the combination of budesonide has been reported to improve the clinical therapeutic effect of azithromycin on MPP in children.^13^ N‐acetylcysteine is a kind of mucolytic agent with a strong phlegm solubilization effect.^14^ The free sulfhydryl group of N‐acetylcysteine can destroy the disulfide bond in the glycoprotein polypeptide chain in sputum, thus cutting down the viscosity of sputum to promote its expulsion.^15^ N‐acetylcysteine can block the initiation of inflammatory cytokines, exert strong antioxidant function, promote the production of lung surfactant, protect alveolar elasticity, and thus improve lung function.^16^, ^17^, ^18^ However, its application in MPP has not been explored.

The present study aimed to explore the therapeutic effect of aerosolized budesonide and N‐acetylcysteine combination therapy for MP infection in children, to propose a more optimized treatment plan for MPP.

## 2 MATERIALS AND METHODS

### 2.1 Study subjects

One hundred and twenty children with MP infection who were admitted to The Second Affiliated Hospital of Qiqihar Medical University from February 2022 to December 2022 were taken as the study subjects. All the children had different degrees of cough, dyspnea, shortness of breath, and other clinical symptoms, auscultation of lung moist rales. MP infection in children was finally diagnosed based on chest X‐ray and positive results of both MP RNA polymerase chain reaction tests of nasopharyngeal secretions and MP‐IgM on serology immediately on admission.^19^ Exclusion criteria: (1) patients with wheezing symptoms; (2) Chronic cough caused by bacteria, viruses, or other pathogens; (3) Patients with serious organic diseases.

### 2.2 Therapeutic schedule

After entering the hospital, two groups of children received general routine treatment measures, including anti‐infection, nutritional support, expectorant, cough treatment, antipyretic, oxygen therapy, and other therapeutic measures. According to the randomized double‐blind method, all cases were divided into a control group and an experimental group, with 60 cases reached. Cases in the control group received aerosol inhalation of budesonide (Pulmicort respules; specification: 2 mL, 0.5 mg/piece, AstraZeneca Pharmaceutical Co.) of 0.25–0.5 mg on the basis of the routine treatment, with the frequency of 10 min/time and twice/day, oxygen flow rate maintains 5 L/min. For children in the experimental group, aerosol inhalation of N‐acetylcysteine solution (Fluimucil; specification: 3 mL, 0.3 g × 5 ampoules/box, Zambon S.P.A.) was added on the basis of the treatment of the control group. The dosage is 3 mL/time, two to three times a day. The treatment continued for 2 weeks.

### 2.3 Observational index

Before treatment and at 3, 5, 7, and 14 days after treatment, the severity of cough in the two groups was assessed based on a day‐time cough symptom scoring system.^20^ The score ranges from 0 to 3, with score of 0 denoting no cough while score of 3 indicates severe cough. The disappearance time of fever, rales, and cough and the occurrence of adverse reactions, including hoarseness and tachycardia, were recorded in the two groups.

### 2.4 Detection of serum inflammatory cytokines

Before treatment and at 3, 7, and 14 days after treatment, 3 mL fasting venous blood was gained from all children. Serum levels of interleukin (IL‐6) and tumor necrosis factor‐α (TNF‐α) were detected by enzyme‐linked immunoassay. The kits were provided by Shanghai Senxiong Company (Shanghai, China). Serum C‐reactive protein (CRP) levels were detected by immune nephelometry according to the manufacturer's instructions.

### 2.5 Clinical efficacy evaluation

Recovery means most of the clinical symptoms such as cough and expectoration disappeared, and recovery was achieved by lung X‐ray examination. Effective means cough, expectoration, and other clinical symptoms turned to a certain extent, and gradually recovered after lung X‐ray examination manifested as lightening or significantly reducing of the shadow of the lungs. Ineffective means cough, sputum, and other clinical symptoms did not improve, even aggravating phenomenon, the lower lobe of the lung still has pathological changes. Total effective rate is the sum of both the recovery rate and the effective rate.

### 2.6 Statistical analysis

The data was imported into the SPSS 22.0 software for calculation. The measurement data were represented by mean value and standard deviation. If the measurement data met the normal distribution, the *t*‐test was adopted, while the rank sum test was selected when not meeting the normal distribution. Counting data were analyzed by chi‐square test.

## 3 RESULTS

### 3.1 General information of two groups of children

The general information of the study population was recorded in Table 1. The age of included children ranged from 2 to 12 years old. Regarding the age and gender, no significant difference was obtained between the control and experimental group, with the median age of 7.02 ± 2.58 and 7.27 ± 2.47, respectively (*p* > .05). Similarly, body mass index distribution also exhibited no significant difference between the two groups (*p* > .05). Based on the chest X‐ray results, most of the children had one lobe infection in each group with no obvious difference between groups (*p* > .05). In addition, some children had a history of rhinitis and atopic dermatitis, but their distribution was not significantly different between the two groups (*p* > .05).

**Table 1:** General information of two groups of children.

Items	Control group	Experimental group	*p* Value
Age, years	7.02 ± 2.58	7.27 ± 2.47	.589
Gender, male/female	27/33	25/35	.713
BMI, kg/m^2^	16.12 ± 1.37	16.05 ± 1.57	.784
Disease course, day	5.17 ± 1.29	4.97 ± 1.21	.382
Lesion area (lung lobes), *n* (%)			.658
One lobe	48 (80.00)	46 (76.67)	
Two or more lobes	12 (20.00)	14 (23.33)	
History of allergic rhinitis, *n* (%)			.570
Yes	8 (13.33)	6 (10.00)	
No	52 (86.67)	54 (90.00)	
History of atopic dermatitis, *n* (%)			.648
Yes	13 (21.67)	11 (18.33)	
No	47 (78.33)	49 (81.67)	

Abbreviation: BMI, body mass index.

### 3.2 Comparison of clinical symptoms between the two groups

**Figure 1:**
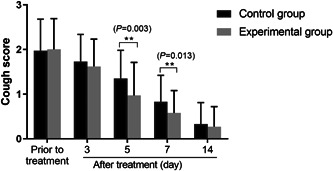
Comparison of cough degree between the two groups. With the passage of treatment time, the children's cough scores of two groups were gradually reduced. **p < .01.

A day‐time cough symptom scoring system was applied for the severity of cough. As displayed in Figure 1, there was no difference for cough scores between the control and experimental groups before treatment (*p* > .05). With the passage of treatment time, the two groups of children's cough scores were gradually reduced. The children in the experimental group got well from the cough faster than the control group, after the treatment of the 5th and 7th days, children's cough score in the experimental group was significantly lower than the control group (*p* < .05).

**Figure 2:**
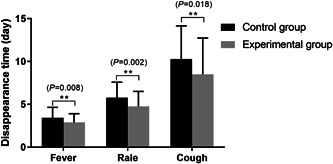
The disappearance time of fever, rale, and cough was compared between the two groups after treatment. **p < .01.

In addition, the disappearance time of clinical symptoms was recorded. As shown in Figure 2, the time required for fever, rale, and cough to disappear in the experimental group was shorter than that in the control group, with a statistically significant difference (*p* < .05).

### 3.3 Comparison of inflammatory cytokines between the two groups

**Figure 3:**
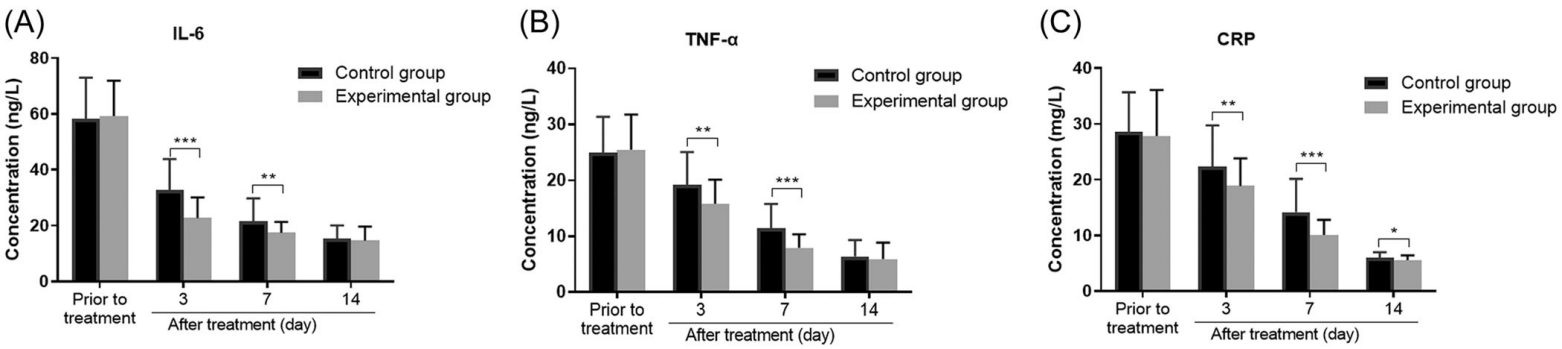
Comparison of inflammatory cytokines between the two groups. On the 3rd and 7th day of treatment, the serum levels of interleukin‐6 (A), tumor necrosis factor‐α (B), and C‐reactive protein (CRP) (C) in the experimental group were significantly lower in the experimental group than those in the control group. **p < .01, ***p < .001.

Furthermore, inflammation‐related cytokines were compared between the two study groups. As seen in Figure 3, serum IL‐6, TNF‐α, and CRP levels showed no obvious difference between the two groups (*p* > .05). As the treatment progressed, we observed a decrease in the levels of three inflammatory cytokines in both groups, and the decline degree was more significant in the experimental group (Figure 3A,B). On the third and 7th day of treatment, the difference met the significant level (*p* < .01). For CRP, levels in the experimental group were significantly lower than those in the control group at all three‐time points after treatment (Figure 3C, *p* < .05).

### 3.4 Comparison of effective rate and adverse reactions between the two groups

Fourteen days after treatment, all children were evaluated. A total of 40 cases in the control group achieved recovery by lung X‐ray, and 16 cases gradually recovered after lung X‐ray examination manifested as lightening or significantly reducing shadow of the lungs. But four patients still had pathological changes in the lower lobe of the lung. In the experimental group, 44 patients achieved recovery by lung X‐ray and 15 cases gradually recovered after lung X‐ray examination manifested as lightening or significantly reducing of the shadow of the lungs, while only one case still had pathological changes in the lower lobe of the lung. As displayed in Table 2, the total effective rate of the experimental group was 98.33%, which surpassed the control group (93.33%), although the difference was significant in statistical analysis.

**Table 2:** Comparison of clinical efficacy between the two groups.

Group	Recovery	Effective	Ineffective	Total effective rate
Control group	40 (66.67)	16 (26.66)	4 (6.67)	56 (93.33)
Experimental group	44 (73.33)	15 (25.00)	1 (1.67)	49 (98.33)
*χ* ^2^				1.895
*p* Value				.169

In the experimental group, nausea occurred in two cases, pharyngeal discomfort in three cases, dizziness in one case, the incidence of adverse reactions was 10.00%. There were three cases of nausea and two cases of pharyngeal discomfort in the control group, and the incidence of adverse reactions was 8.33%. The incidence of adverse reactions in the experimental group was slightly higher than that in the control group, but no statistically significant difference was detected between the groups (*χ* ^2^ = 0.100, *p* > .05).

## 4 DISCUSSION

MPP is a common respiratory disease in children with a long incubation period. MPP tends to occur in school‐age children, the incidence has also increased in children under 5 years old in recent years.^21^ The early symptoms of MPP are characterized by fever, anorexia, cough, and, as the disease progresses, persistent fever, dyspnea, or respiratory distress, and in severe cases, death.^22^ The clinical treatment of MPP in children is mainly comprehensive treatment, including antibiotics, hormones, and so on.^23^ As a third‐generation macrolide antibiotic, azithromycin can rapidly bind to bacterial ribosome subunits, inhibit their protein synthesis, and produce antibacterial effects.^24^ However, azithromycin alone is difficult to rapidly relieve symptoms such as cough and asthma in the treatment of mycoplasma pneumonia.^25^, ^26^ Therefore, it is driven that azithromycin serves as the basic treatment against pathogens, combined with other drugs to optimize the therapeutic effect and improve clinical efficacy. In this study, the combined use of budesonide and N‐acetylcysteine on the basis of conventional azithromycin is effective.

Damage to alveolar epithelium during pulmonary MP infection leads to the activation of alveolar macrophages.^27^ Indeed, when activated, immune cells including macrophages and lymphocytes play a crucial role in inflammatory responses through the release of various endogenous mediators such as pro‐inflammatory prostaglandins. TNF‐α and IL‐6 are two well‐known pro‐inflammatory cytokines that are thought to be central mediators in the cytokine cascades, and also important cytokines for immune and inflammatory response.^28^ Overexpression of IL‐6 can cause damage to multiple organs and systems.^29^ TNF‐α, which is mainly derived from macrophages, has a wide range of biological and immune functions. TNF‐α can directly damage lung endothelial cells and cause lung tissue injury.^30^ A large number of studies have shown that high expression of TNF‐α was detected in mononuclear macrophages, adhesion cells and lymphocytes of the host lung during MPP, suggesting that TNF‐α was involved in the pathogenesis of MPP.^31^, ^32^ The results of this study showed that serum levels of IL‐6 and TNF‐α were significantly increased in children with MP, suggesting that IL‐6 and TNF‐α, as important pro‐inflammatory factors, are involved in the pathological process of lung inflammation and play an important role in the occurrence and development of MPP. CRP is an acute reactive protein produced by the liver in response to stress.^33^ It is considered to be a good indicator of inflammation and infection and clinical efficacy.^34^ The present findings supported that serum CRP level was significantly increased in children with MP infection, indicating that CRP level reflects the degree of inflammation damage caused by MP infection to the body to a certain extent. According to the present results, serum TNF‐α, IL‐6, and CRP levels decreased remarkably after treatment, revealing the recovery of inflammatory response of MP‐infected cases caused by budesonide and N‐acetylcysteine. Moreover, inflammatory cytokines fell faster in the experimental group than in the control group, indicating that budesonide and N‐acetylcysteine combination therapy can suppress the inflammatory response more quickly, the anti‐inflammatory effect is significant. It is reported that budesonide can limit the excessive stimulation of immune cells, as well as the release of inflammatory cytokines.^35^ N‐Acetylcysteine has shown the potential to improve the immune state of patients by downregulating pro‐inflammatory through modulating and suppressing the NLRP3 inflammasome pathways of macrophages.^36^ Our present results also determined the regulatory role of budesonide and N‐acetylcysteine in the immune function of MP infection.

Clinically, glucocorticoid is commonly used for MPP treatment and the improvement of inflammatory response.^23^ In this study, the clinical treatment of budesonide combined with N‐acetylcysteine by aerosol inhalation was effective. Aerosol inhalation of therapeutic drugs can directly act on bronchial mucosa, promote continuous local precipitation of drugs, prolong anti‐inflammatory time, and avoid liver first‐pass effect, which can help to improve airway inflammation quickly.^37^ Budesonide is a glucocorticoid drug, which can rapidly play the anti‐immune function and anti‐inflammatory effect.^38^ It has been widespreadly applied in the clinical treatment of pediatric pneumonia.^39^ Acetylcysteine has certain antioxidant damage, and it is rich in free sulfhydryl (‐SH) groups, which can break the mucin disulfide bond in sputum, reduce the viscosity of sputum, promote sputum discharge as soon as possible, and quickly relieve the cough and sputum symptoms of children.^40^ A recent clinical study has reported that N‐acetylcysteine combination can improve the treatment of bronchopneumonia in children and quick relief of lung rales.^41^ Consistently, the results of this study suggested that budesonide and N‐acetylcysteine combination therapy can accelerate the relief of clinical symptoms in MP‐infected children, including fever, rale, and cough. Furthermore, a high effective rate was found in cases that received budesonide and N‐acetylcysteine combination therapy, although the difference was not significant. The reason might be the small sample size. It was concluded that budesonide and N‐acetylcysteine combination therapy can quickly relieve the clinical symptoms of MP‐infected children although it did not significantly improve the cure rate.

In conclusion, budesonide and N‐acetylcysteine combination therapy in the treatment of MP infection in children has a significant effect and can quickly relieve the clinical symptoms of children with good safety. It is worthy of widespread clinical use.

## AUTHOR CONTRIBUTIONS

**Jing Chen**: Conceptualization; formal analysis; methodology; writing—original draft. **Ying Zhu**: Data curation; investigation; software. **Chunfeng Zheng**: Investigation; methodology; validation. **Wei Zhao**: Data curation; methodology; validation. **Qi Liu**: Conceptualization; project administration; supervision; writing—review and editing.

## CONFLICT OF INTEREST STATEMENT

The authors declare no conflict of interest.

## ETHICS STATEMENT

The study was advanced with the approval of the Second Affiliated Hospital of Qiqihar Medical University Ethics Committee. Informed consent was obtained from legal guardians.

## Data Availability

All data generated or analyzed during this study are included in this article. Further enquiries can be directed to the corresponding author.
